# Development of an active packaging system containing zinc oxide nanoparticles for the extension of chicken fillet shelf life

**DOI:** 10.1002/fsn3.1812

**Published:** 2020-08-31

**Authors:** Azam Ahmadi, Parisa Ahmadi, Ali Ehsani

**Affiliations:** ^1^ Student Research Committee Tabriz University of Medical Sciences Tabriz Iran; ^2^ Department of Food Sciences and Technology Faculty of Nutrition and Food Sciences Tabriz University of Medical Sciences Tabriz Iran; ^3^ Food and Drug Safety Research Center Tabriz University of Medical Science Tabriz Iran

**Keywords:** antimicrobial activity, bacterial inoculation, bionanocomposite film, cellulose nanofibers, gelatin, ZnO NPs

## Abstract

The casting method was employed to prepare gelatin‐based nanocomposite films containing different concentrations of cellulose nanofiber (CNF) as a reinforcement filler (2.5%, 5%, and 7.5% w/w of gelatin) as well as zinc oxide nanoparticles (ZnO NPs) as an antimicrobial agent (1%, 3%, 5%, and 7% w/w of gelatin). The results showed that the incorporation of 5% CNFs (optimum concentration) significantly boosted the films' stiffness (YM; by 47%) and strength (TS; by 72%) but decreased its flexibility (EAB; by 28%), water vapor permeability, and moisture absorption. The best G/CNF film antibacterial activity was provided by the 5% concentration of ZnO NPs according to the disk diffusion assay; Gram‐positive bacteria were inhibited significantly more than Gram‐negative bacteria. The antimicrobial efficacy of the G/CNF/ZnO NPs film as a food packaging material was assessed via counts of *Staphylococcus aureus* and *Pseudomonas fluorescens* inoculated on chicken fillets (as a food model) in the treatment (G/5% CNF/5% ZnO) and control groups (plastic bag). The antibacterial film led to a significant reduction in the bacterial load of the chicken fillets (*p* < .05), especially against the Gram‐positive strain. This study illustrated that G/CNF/ZnO NPs films can be utilized as active packaging to prolong the shelf life of different perishable foods such as meat.

## Graphical abstract

The optimum concentrations of CNF (5% from physicomechanical characteristics and water resistance) and ZnO NPs (5% from the disk diffusion method) were selected as a food packaging for counts of *Staphylococcus aureus* and *Pseudomonas fluorescens* inoculated on chicken fillets.

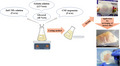

## 1 INTRODUCTION

In general, food packaging is aimed at preventing food degradation caused by physical and chemical processes or microbial contamination, thereby reducing aroma loss and maintaining product quality during its extended shelf life. To achieve this, the movement of water and gases must be restricted by the packaging material, which must simultaneously conform to the physicomechanical requirements (Talegaonkar, Sharma, Pandey, Mishra, & Wimmer, 2017). The petrochemical polymers currently utilized as food packaging are nonrenewable and nonbiodegradable, presenting remarkable threats such as natural resource depletion, energy crises, global warming, and ecological problems pertaining to waste generation and disposal (Sarwar, Niazi, Jahan, Ahmad, & Hussain, 2018). In recent decades, environmental concerns and increasing consumer demand for healthy and nutritious food products with prolonged shelf life have made the food and packaging industries pay more attention to the preparation of edible, biodegradable packaging films from natural macromolecules and biopolymers such as proteins, polysaccharides, and lipids or their combinations (Mohammadi, Kamkar, & Misaghi, 2018; Pirouzifard, Yorghanlu, & Pirsa, 2019).

Gelatin is one of the most common biopolymer proteins, obtained from thermal hydrolysis or physicochemical destruction of collagen derived from animal skin and bone (Núñez‐Flores et al., 2012). The low cost, efficient lipid barrier properties, film‐forming capacity, and biodegradability of gelatin make it suitable for use in edible film production. However, its weak mechanical properties, inadequate bonding capacity, and high permeability to water are its major drawbacks (Bodini, Sobral, Fávaro‐Trindade, & Carvalho, 2013). Thus, suitable fillers can be added to gelatin films to form composites with amended properties. Nanocomposites illustrate a promising alternative for achieving improved physicomechanical, thermal, and water resistance properties (Arora & Padua, 2010).

Cellulose nanofibers (CNFs) are aggregations of primary cellulose fibrils with lengths of 2–20 nm in diameter isolated through mechanical operations, such as high‐pressure homogenization, grinding, and purifying (Chen et al., 2011). As highly applicable organic nanofillers for the reinforcement of nanocomposite films, CNFs are biodegradable, abundant, accessible, nontoxic, lightweight and have high aspect ratios (Deepa et al., 2016). Studies on CNFs have shown that they can improve the mechanical performance of films and reduce their water solubility, swelling ratio, and water vapor permeability (Chaabouni & Boufi, 2017; Narita, Okahisa, & Yamada, 2019; Samadani, Behzad, & Enayati, 2019; Yu et al., 2017). As an effective reinforcement additive for biopolymer materials, CNF backbone chains have a unique intrinsic structure that facilitates interfacial interactions between the polymer matrix and the nanoparticles (Dai et al., 2017).

On the other hand, microbial contamination is a long‐standing problem affecting foods (particularly meats) since foodborne bacteria and fungi are involved in food spoilage and poisoning, leading to economic losses, human health risks, quality reduction, and decreased product life (Clarke et al., 2016; Seydim & Sarikus, 2006). Among the various meats, chicken is more extensively consumed considering it is low‐fat, nutritious and has a relatively low price (Azlin‐Hasim et al., 2016). Notwithstanding all the mentioned advantages, this meat is highly susceptible to spoilage as its protein and moisture components, as well as suitable pH, allow the growth of both pathogenic and nonpathogenic microbes (Takma & Korel, 2019). The use of metal oxide nanoparticles such as TiO2, ZnO, and CuO as thermally stable antimicrobial agents for the production of active biopolymer packaging is one of the ways of preventing foodborne illness and thereby enhancing food safety (McMillin, 2017; Shankar, Teng, & Rhim, 2014). An example of these metal oxides is zinc oxide NPs (ZnO NPs), which have a crystalline structure, an extensive surface area, low cytotoxicity, semiconductor behavior, and excellent mechanical, antimicrobial, thermal, and UV light barrier characteristics (Pirsa & Shamusi, 2019; Shankar, Teng, Li, & Rhim, 2015).

In the present research, we aimed to fabricate G/CNF films via the casting method to achieve improved physicomechanical properties relative to neat gelatin. Then, we developed active antimicrobial films by adding ZnO NPs to the G/CNF film and evaluated the antimicrobial properties of the product using chicken fillet as a food model.

## 2 MATERIALS AND METHODS

### 2.1 Materials

Gelatin (60 kDa; 225 g Bloom gel strength; cat. no. 1.04078.1000) was obtained from Merck Co. Cellulose nanofibers (CNFs; white gel 2.5%; ~45 nm mean diameter, ~78% crystallinity, ~99% pure) were supplied by Nano Novin Polymer Co., whereas the ZnO NP powder (zinc oxide; 10–30 nm; >99% pure) was purchased from US Research Nanomaterials Inc. Culture media including Cetrimide Fucidin Cephaloridine (CFC) agar, Baird‐Parker agar (BPA), nutrient agar and broth, and Mueller‐Hinton agar were provided by Micromedia. Only reagents with analytical grade were used.

#### 2.1.1 Bacterial strains

The Biological and Genetic Resources Center supplied the *Staphylococcus aureus* (ATCC 33591) and *Pseudomonas fluorescens* (ATCC 13525) strains.

### 2.2 Methods

#### 2.2.1 Preparation of G/CNF and G/CNF/ZnO nanocomposite films

To prepare the films, we utilized the casting procedure. The gelatin film solution (as control) was made by mixing gelatin powder (3.5% w/w) in distilled water and heating the mixture with a water bath (JulaboMP‐5) at 70°C for 1 hr until obtaining a clear, light yellow solution. To prepare the CNF suspension, different concentrations of CNF gel (2.5%, 5%, and 7.5% based on gelatin) were dispersed in distilled water with continuous stirring for 2 hr. To facilitate complete dispersion, an ultrasonic bath treatment (Belfor) was applied for half an hour. Then, the CNF suspensions were dropped into the gelatin solution. Then, as the plasticizer, 40% glycerol (based on gelatin) was added to prepare G/CNF nanocomposite films under stirring. ZnO NP solutions (1%, 3%, 5%, and 7% w/w based on gelatin) were prepared by the same production procedure of the CNF suspensions, before being added drop by drop to the G/CNF 5% film solution containing 1.4 g of glycerol under continuous stirring over 2 hr. The film solutions were then spread within Petri dishes (diameter = 8 cm) and left to dry at ambient temperature for 48 hr. Subsequently, the dry film samples were separated and kept in polyethylene bags.

### 2.3 Characterization of G/CNF films

#### 2.3.1 Determination of mechanical properties

To evaluate the films' mechanical characteristics, including Young's modulus (YM), percentage of elongation at break (EAB), and tensile strength (TS), the standard methods of the ASTM (Standard, 2010) were followed using the Sanaf Universal Testing Machine (Tehran, Iran). The film specimens, after being conditioned for 24 hr at 55 ± 3% RH and 25°C, were cut into dumbbell shapes and loaded into the device (50 mm initial grip separation; 10 mm/min crosshead speed; 25 N load; room temperature).

#### 2.3.2 Film thickness

A digital micrometer (Guanglu; 0.01 mm precision) was used to evaluate the thickness of each film sample at five different points that were randomly selected. The mean value was reported.

#### 2.3.3 Light transmittance and opacity

To investigate the film's optical barrier characteristics, a spectrophotometer (Unico, UV‐2100) was employed with a wavelength range of 400–800 nm. Rectangular film samples (1 × 5 cm) were used, with an empty cell being the point of reference. Equation 1 was used to calculate the opacity of the films (Salari, Khiabani, Mokarram, Ghanbarzadeh, & Kafil, 2018).

(1) $$\text{Opacity}\;=\;\frac{\text{Abs600}}{X}$$

Here, Abs_600_ is the absorbance at 600 nm and *X* denotes the thickness of the film in mm.

Higher opacity is indicative of lower light transmission.

#### 2.3.4 Moisture content

To determine the films' moisture content (MC), the gravimetric procedure was employed. Film strips (20 mm × 20 mm) were dried within a laboratory oven at 105 ± 1°C until reaching steady weights. After triplicate experimentation, the mean weight values were inserted into Equation 2 to obtain the MC.

(2) $$\text{MC}\;\left(\%\right)\;=\;\frac{M_{0}‐M_{1}}{M_{0}}\;\times \;100$$

Here, *M*_0_ and *M*_1_ represent the film weight (g) initially and postdrying, respectively (Salari et al., 2018).

#### 2.3.5 Moisture absorption (MA)

Evaluation of the films' capacity to absorb moisture was performed according to the method of Almasi, Ghanbarzadeh, Dehghannya, Entezami, and Asl (2015) with slight modifications. Film strips (20 × 20 mm^2^) were subjected to conditioning with CaCl_2_ (0% RH) at room temperature. The samples were then weighed before being placed in a desiccator containing saturated sodium chloride solution (75% RH). The weighing was repeated multiple times until an equilibrium state was achieved. The MA was obtained using Equation 3.

(3) $$\text{MA}\;\left(\%\right)\;=\;\frac{W_{t}‐W_{0}}{W_{0}}\;\times \;100$$

In the above equation, the initial sample weight (at 0% RH) and the weight at time *t* (at 75% RH) are represented by *W*_0_ and *W_t_*, respectively.

#### 2.3.6 Water solubility (WS)

To evaluate the solubility of the film samples with respect to water, the procedure described by Hosseini, Rezaei, Zandi, and Farahmandghavi (2015) was slightly modified and employed. Three pieces (2 × 2 cm^2^) of the films were dried for 6 hr in a laboratory oven (105°C). The initial weight (*W*_i_) of the films was then recorded (±0.0001 g). Subsequently, the films were immersed in distilled water (50 ml) then gently shaken at 100 rpm overnight at ambient temperature. Preweighed filter paper was then used for sample filtration. The insoluble section and filter paper were subjected to oven‐drying (105°C, 6 hr) before the final weight was recorded (*W*_f_) and WS% was obtained by use of Equation 4.

(4) $$\text{WS}\;\left(\%\right)\;=\;\frac{W_{i}‐W_{f}}{W_{i}}\times 100$$

#### 2.3.7 Determination of water vapor permeability (WVP)

To determine the films' WVP, the standard ASTM E96‐05 (ASTM, 2005) procedure was followed. Cylindrical vials containing 5 g of anhydrous calcium sulfate (CaSO_4_, RH = 0%) were used as the permeation cells. The conditioned round‐shaped film pieces were used to seal the vials, before the initial weight was recorded. The sealed vials were then placed in a desiccator containing distilled water (RH = 100%) and set at room temperature. The vials were repetitively weighed at 1 hr intervals over 8 hr. After plotting the weight as a function of time (weight vs. time) and determining the slope by linear regression (*r* ^2^ > .99), Equations (5), (6), (7) were applied to determine the WVP (g.mm/Pa.h.mm^2^), water vapor transmission rate (WVTR; g/h.mm^2^), and partial water vapor pressure difference across the film (ΔP; Pa).

(5) $$\text{WVP}\;=\;\frac{\text{WVTR}\;\times \;L}{\Delta P}$$

(6) $$\text{WVTR}\;=\;\frac{\text{slop}}{A}$$

(7) $$\Delta P\;=\;P\;\left(H_{1}‐H_{2}\right)$$

In the above equations, the thickness (mm) of the films is denoted by *L*; *P* is the vapor pressure of water at the saturation point (3,169 Pa) and room temperature, with *H*_1_ and *H*_2_ denoting the relative humidity within the desiccator and vial, respectively.

### 2.4 Scanning electron microscopy (SEM)

A TESCAN MIRA 3 XMU scanning electron microscope (SEM) was used to investigate the influence of the CNFs and ZnO NPs on the morphological characteristics of the gelatin‐based films. For this purpose, the film samples were sputter‐coated with gold; the acceleration voltage used during scanning was 10 kV.

### 2.5 Fourier transform infrared (FT‐IR) spectroscopy

To analyze the chemical structure and interactions between the components, FT‐IR spectroscopy was applied in the range of 4,000–500 cm^−1^; and 100 scans were performed with a resolution of 1 cm^−1^. The FT‐IR spectra of the gelatin, G/CNF, and G/CNF/ZnO films in KBr pellets were measured on a Bruker Tensor‐27 Spectrometer at room temperature and reported based on the transmission.

### 2.6 Microbial characterization

#### 2.6.1 Evaluation of the antibacterial activity of G/CNF/ZnO films

The activity of the nanocomposites against bacteria was evaluated by the disk diffusion test. *Pseudomonas fluorescens* and *Staphylococcus aureus* were cultured in nutrient broth for 18 hr. The bacterial suspensions were then collected and set to 0.5 McFarland standard turbidity (1.5 × 10^8^ CFU/ml). Next, through 1:100 dilutions, bacterial densities of 1.5 × 10^6^ CFU/ml were achieved. Next, the nanocomposite films were cut into pieces with 6 mm diameter under sterile conditions and placed on the surface of Mueller‐Hinton agar plates; 100 μl aliquots of the prepared suspensions were inoculated ahead of incubation, which was done overnight at 37 and 25°C for *S. aureus* and *P. fluorescens*, respectively. The zone of inhibition surrounding each disk was then evaluated with a digital micrometer, and the film with optimal antimicrobial properties was chosen for covering chicken fillet samples.

#### 2.6.2 Chicken fillet samples preparation and treatment

The samples of fresh chicken fillets were purchased locally. After immediately being taken to the laboratory, the samples were washed and cut aseptically into squared pieces weighing 10 g. After sterilizing the pieces using ethanol (95% v/v) and UV light, they were inoculated with 10^4^ CFU/g of *P. fluorescens* and *S. aureus* suspensions. The samples were divided into the treatment (packaged with the optimized G/5% CNF/5% ZnO film) and control (packaged with sterile transparent plastic polyethylene) groups. Storage occurred at the temperature of 4 ± 1°C over 12 days; microbial characteristics were evaluated initially and then at 3‐day intervals.

#### 2.6.3 Microbial evaluation of bacteria inoculated in chicken fillets

To conduct the microbial analysis, chicken fillet samples weighing 10 g were blended for 5 min with 0.1% sterile peptone water (90 ml) with a Stomacher 400 device (Seward Medical). Then, 100 μl serial dilutions (10^−1^ to 10^−8^) were spread on pre‐prepared Baird‐Parker (BP) agar for *S. aureus* and Cetrimide Fucidin Cephaloridine (CFC) agar for *P. fluorescens*. Incubation occurred at 25 and 37°C for *P. fluorescens* and *S. aureus*, respectively.

### 2.7 Statistical analysis

The SPSS 21.0 software (IBM) was utilized for statistical analysis, with values being presented as mean ± standard deviation (*SD*). All tests were performed in triplicates. The independent *t* test, analysis of variance (ANOVA), and Tukey's post hoc test were conducted; significance was regarded at *p* < .05.

## 3 RESULTS AND DISCUSSION

### 3.1 Thickness

The thickness of a film is influenced by the incorporation of fillers into its matrix. Table 1 reveals that the thickness of the neat gelatin film was 0.102 ± 0.005 mm; this value increased to 0.11 ± 0.006 mm after incorporation of up to 7.5% CNFs. Similar results were reported in whey protein isolate‐based films when adding CNFs (Alizadeh‐Sani, Khezerlou, & Ehsani, 2018).

**TABLE 1:** The physical properties of gelatin films incorporated with cellulose nanofibers

Samples	WVP (×10^−10^ g.mm/Pa.h.mm^2^)	MC (%)	WS (%)	MA (%)	Thickness (mm)
Gelatin	12.95 ± 0.70^a^	7.94 ± 0.44^a^	59.83 ± 1.11^a^	8.30 ± 0.06^a^	0.102 ± 0.005^a^
G/CNFs 2.5%	12.62 ± 0.42^a^	7.95 ± 0.22^a^	60.00 ± 0.42^a^	7.74 ± 0.34^b^	0.106 ± 0.007^b^
G/CNFs 5%	11.87 ± 0.88^b^	7.92 ± 0.33^a^	59.82 ± 0.27^a^	7.46 ± 0.38^c^	0.108 ± 0.004^b^
G/CNFs 7.5%	12.01 ± 0.35^c^	8.13 ± 0.54^b^	57.45 ± 0.44^b^	6.74 ± 0.24^d^	0.11 ± 0.006^c^

Note

Values represent the mean ± standard deviation of three replicates. Different lowercase letters indicate significantly differences (*p* < .05) between all samples in a column.

Abbreviations: CNFs, cellulose nanofibers; G, gelatin; MA, moisture absorbance; MC, moisture content; WS, water solubility; WVP, water vapor permeability.

### 3.2 Light transmittance and opacity

Film transparency has significance as it directly affects the appearance of coated products, the rate of lipid oxidation, and the quality of the packaged food product. The light (400–800 nm) transmission characteristics of the gelatin‐based films were evaluated via UV–vis spectrophotometry. According to the results (Table 2), the highest light transmittance was observed in the pure gelatin film. The G/CNF composite films became significantly less (*p* < .05) transparent as the nanocellulose content increased. In the G/CNF composite films, light transmission at 800 nm decreased from 76.02 ± 0.30% to 64.03 ± 0.61% when the CNF component was increased from 0 to 7.5 wt%. The film opacity significantly increased to 1.603 ± 0.057 with 7.5% CNF incorporation (*p* ˂ .05), indicating decreased transparency. It has been reported that the addition of nanocellulose to proteins causes light transmittance to be lost mostly as a result of refraction/reflection occurring at the interface of the two species, resulting in increased film opacity (Liu, Tang, & Liu, 2015). The findings concur with those of Alizadeh‐Sani et al. (2018), who found that whey protein film transparency decreased with the addition of CNF. Also, a reduction in film transparency with the incorporation of nanocellulose was observed in whey protein isolate‐nanocellulose bionanocomposite films (Qazanfarzadeh & Kadivar, 2016).

**TABLE 2:** Light transmission percentage and opacity of gelatin film combined with cellulose nanofibers

Samples	Light transmission (%) at different wavelengths (nm)					Opacity 600 nm Mean ± *SD*
	400 Mean ± *SD*	500 Mean ± *SD*	600 Mean ± *SD*	700 Mean ± *SD*	800 Mean ± *SD*	
Gelatin	71.44 ± 0.65^a^	75.40 ± 0.55^a^	75.44 ± 0.20^a^	76.13 ± 0.54^a^	76.02 ± 0.30^a^	1.133 ± 0.005^a^
G/CNFs 2.5%	64.99 ± 0.91^b^	72.17 ± 0.55^a^	73.47 ± 1.07^a^	73.63 ± 0.53^a^	74.50 ± 0.57^a^	1.310 ± 0.020^b^
G/CNFs 5%	60.97 ± 2.00^c^	67.49 ± 1.35^b^	70.43 ± 1.99^b^	70.16 ± 2.33^b^	70.96 ± 2.34^b^	1.510 ± 0.026^c^
G/CNFs 7.5%	56.29 ± 2.91^d^	61.00 ± 2.31^c^	63.27 ± 2.36^c^	64.76 ± 2.15^c^	64.03 ± 0.61^c^	1.603 ± 0.057^d^

Note

Statistical analysis reported based on the ANOVA test. Values represent as the mean ± standard deviation of three replicates. Values with different superscripts were significantly different (*p* < .05).

Abbreviations: CNFs, cellulose nanofibers; G, gelatin.

### 3.3 Water resistance

#### 3.3.1 Moisture content (MC)

The MC is the amount of water “bound or confined” within a sample. According to Table 1, the MC of the films stayed constant with the incorporation of 2.5% and 5% CNFs, whereas the CNF concentration of 7.5% induced a rise from 7.94 ± 0.44% net gelatin to 8.13 ± 0.54% (*p* < .05). This can be attributed to the decreased water binding ability that ensues cross‐linking within the nanocomposite films and the trapping of free water molecules via the created network by the main components of film (Yu et al., 2018). The presence of a large number of free OH groups in the gelatin matrix resulted in a relatively high MC value in neat gelatin film (Wang et al., 2018).

#### 3.3.2 Moisture absorption (MA)

The pure gelatin film absorbed the highest amount of moisture after 24 hr (8.30 ± 0.06%), probably because of its hydrophilic amino acids. By adding 2.5% to 7.5% of CNFs, the MA significantly decreased from 7.74 ± 0.34% to 6.74 ± 0.24% (*p* < .05) (Table 1). Despite the inherently hydrophilic nature of the CNF, its stiffness and crystallinity impede its ability to absorb water and reduce the porosity of the films. This phenomenon may also be explained by the development of electrostatic interactions and hydrogen bonding between the protein's amine groups and the OH groups present in the CNFs, decreasing the capacity for hydrogen bonding with water molecules (Ranjbaryan, Pourfathi, & Almasi, 2019; Samadani et al., 2019). This reducing trend was also reported in carboxymethyl cellulose‐based (Zabihollahi, Alizadeh, Almasi, Hanifian, & Hamishekar, 2020) and chitosan‐based films (Soni, Schilling, & Mahmoud, 2016).

#### 3.3.3 Water solubility (WS)

Table 1 reveals the findings pertaining to the solubility of the films within water. In the composite samples, no obvious changes in WS were observed with the addition of up to 5% CNFs (*p* > .05), meaning that the gelatin film was left largely unaffected in terms of water resistance. However, increasing the concentration of CNFs up to 7.5% led to decreased WS from 59.83 ± 1.11% to 57.45 ± 0.44% in comparison with the neat gelatin film (*p* < .05). These results confirm those of previous studies on alginate and whey protein nanocomposite films (Abdollahi, Alboofetileh, Rezaei, & Behrooz, 2013; Alizadeh‐Sani et al., 2018).

#### 3.3.4 Water vapor permeability (WVP)

For food packaging, water vapor permeability is affected by two factors: the solubility and diffusion of water molecules (Pirsa, Karimi Sani, & Khodayvandi, 2018). Since moisture in food accelerates microbial and chemical spoilage, moisture must be prevented from reaching foods (Ahmadi et al., 2019). According to WVP results shown in Table 1, incorporation of CNFs caused a significant drop in WVP from 12.95 ± 0.70 (×10^−10^ g.mm/Pa.h.mm^2^) in the neat gelatin film to 12.01 ± 0.35 (×10^−10^ g.mm/Pa.h.mm^2^) in the G/7.5% CNF film. It is noteworthy that the lowest water permeation was for films containing 5 wt. % CNFs (11.87 ± 0.88 × 10^−10^ g.mm/Pa.h.mm^2^). The significant impact of CNFs on reducing the WVP of PLA (Almasi et al., 2015), starch (Li, Tian, Jin, & Li, 2018), gluten (Bagheri et al., 2019), and sodium caseinate films (Ranjbaryan et al., 2019) has been reported in the literature. The decreased WVP in the G/CNF films may be due to the positioning of the CNF chains within the matrix of the film and the development of hydrogen bonds between gelatin and CNF hydroxyl groups, thereby improving the matrix cohesion. Also, the creation of a long, zigzag, turbulent pathway against the transfer of water vapor decreased the number of hydrophilic groups (‒OH) and adsorbed water molecules. Tortuous paths pertain to the weight ratio, shape, and dispersion of the reinforcement material, matrix and filler adherence, polymeric chain mobility, and composite porosity (Song, Xiao, & Zhao, 2014). Filling the void space between the biopolymer chains with CNFs reduces chain mobility and reduces the diffusion rate of water molecules (Ahmadi et al., 2019). At CNF concentrations above 5%, the dispersion weakened probably due to the aggregation of nanofibers, and the WVP increased as more pores were formed (Bagheri et al., 2019).

### 3.4 Mechanical properties

**FIGURE 1:**
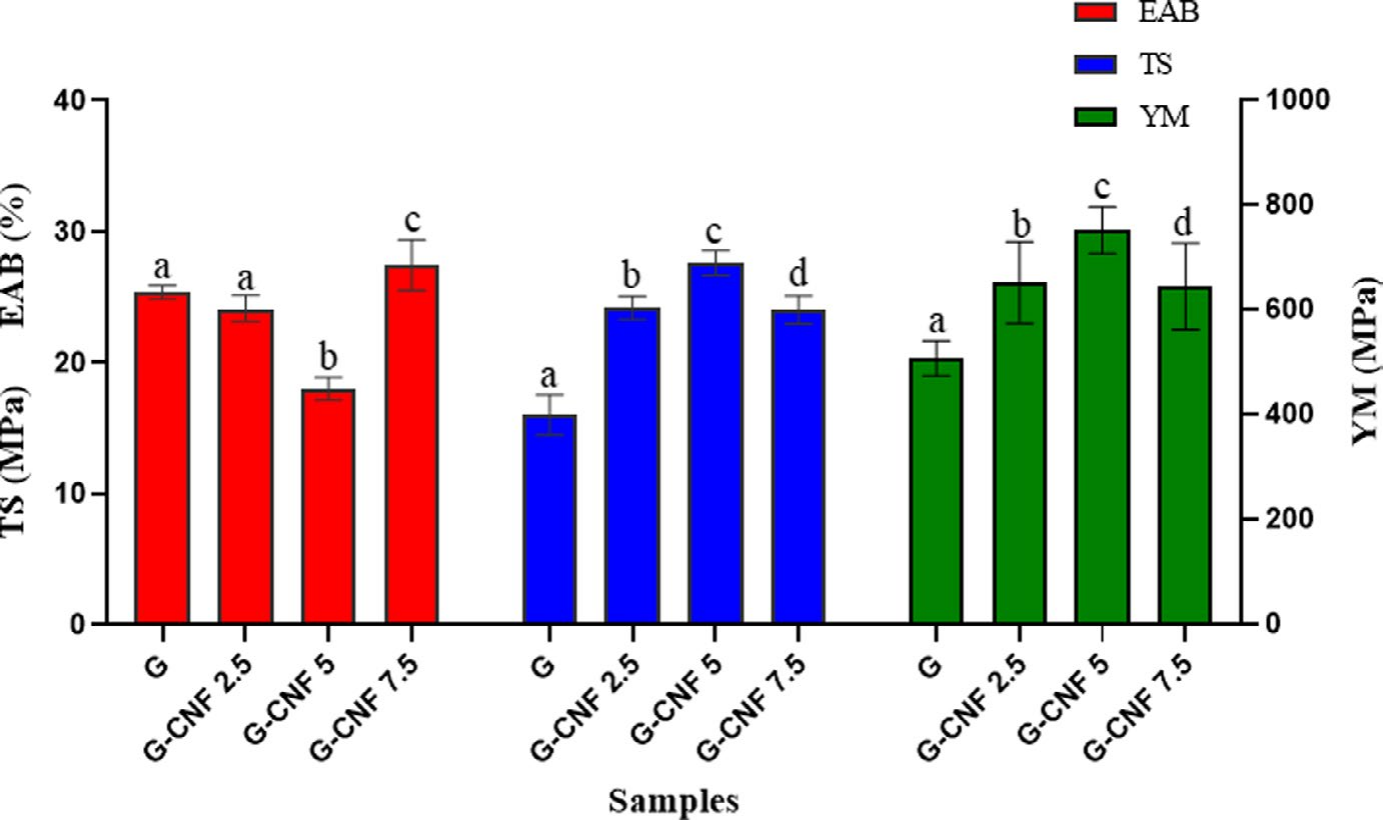
The mechanical parameters of G/CNF nanocomposite films. CNFs, cellulose nanofibers; G, gelatin

Extensibility and mechanical strength are typically required for the maintenance of the physical integrity and barrier properties of film materials in the face of external forces in food packaging applications (Souza et al., 2012). The influence of CNFs on the films' mechanical characteristics is presented in Figure 1. The TS, EAB, and YM values for the neat gelatin film were 16.02 ± 1.54 MPa, 25.37 ± 0.52%, and 508.95 ± 33.58 MPa, respectively. With the addition of 5% of CNFs, the TS (27.62 ± 0.94 MPa) and YM (752.8 ± 44.54 MPa) values increased significantly (*p* < .05), whereas the EAB (18.02 ± 0.87%) dropped in comparison with the control film (gelatin). This increment in the first two parameters can be attributed to the strong and highly stiff nature of the CNF chains, the high compatibility of fillers in the biopolymer matrix, the uniform dispersion of nanofillers in the polymeric network, the high surface ratio, and the development of a firm, uninterrupted, three‐dimensional G‐CNF network via hydrogen bonds (Alizadeh‐Sani et al., 2018; Majer, Hutař, & Nahlik, 2013). Furthermore, the development of increased G‐CNF interactions and the reduced protein chain mobility results in increased TS but reduced flexibility. The rise in EAB may be attributed to the reinforcement of the soft gelatin biopolymer with rigid nanofillers, resulting in increased hardness. However, it had a brittleness effect on bionanocomposite film compared with pure biopolymer (Alizadeh‐Sani et al., 2018; Wang, Liu, et al., 2017). A similar mechanism was reported for sodium caseinate in a previous study (Ranjbaryan et al., 2019). According to the literature, CNFs improved the mechanical properties of different matrices such as banana starch (Tibolla et al., 2019), carboxymethyl cellulose (Zabihollahi et al., 2020), and triacetate cellulose (Wu, Danh, & Nakagaito, 2020). Nonetheless, contrasting results manifested at CNF concentrations above 5 wt. %. The composite film incorporated with 7.5% of CNFs had decreased TS (24.045 ± 1.06 MPa) and YM (645.6 ± 82.59 MPa) values relative to the aforementioned samples. These findings may be ascribed to the agglomeration of the CNFs with heterogeneous dispersion in the polymeric matrix beyond a particular concentration, which acts as a stress concentration point and destroys the structural integrity of the polymeric matrix, thereby weakening the tensile properties of the films. Similar results have been reported by other authors (Salari et al., 2018; Sarwar et al., 2018).

According to the results of the physicomechanical tests performed in the above sections, the concentration of 5% nanocellulose was selected as the optimal concentration for the next steps.

### 3.5 Antibacterial activity of G/CNF/ZnO nanocomposite films

**FIGURE 2:**
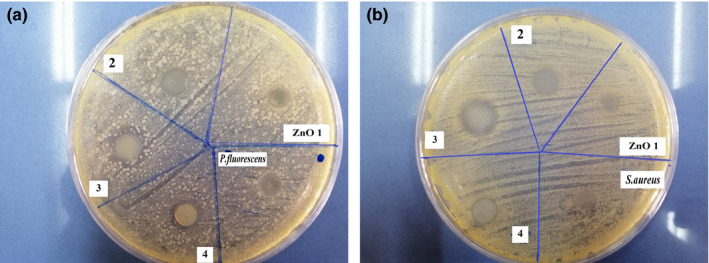
The antibacterial activity of the film samples (G/CNFs/ZnO NPs) against *S. aureus* (a) and *P. fluorescens* (b) after 24 hr of incubation. CNFs, cellulose nanofiber; ZnO NPs, zinc oxide nanoparticles

Active ingredients such as antioxidants or antimicrobials in packaging films can preserve food quality and safety (Takma & Korel, 2019). To provide antimicrobial protection for chicken meat, ZnO NPs were incorporated into G/CNF edible packaging films as an active agent. Figure 2 depicts the results of the disk diffusion assay; the inhibition zones related to the Gram‐negative (*P. fluorescens*) and Gram‐positive (*S. aureus*) strains are shown in Table 3. No clear inhibition zones were found in the G/CNF film (control). As mentioned, CNFs were used to expand the physicomechanical properties of the gelatin film. Antibacterial activity against both species was seen in all active nanocomposites containing ZnO NPs; the sizes of the inhibition zones increased as the ZnO NP concentration increased from 1% to 5% of the polymer. The greatest zone of inhibition against both bacteria was seen in nanocomposites containing 5% ZnO NPs (10.44 ± 0.44 mm and 9.75 ± 0.11 mm for *S. aureus* and *P. fluorescens*, respectively). It has been reported that the antimicrobial activity of ZnO NPs against microorganisms is related to the production of different reactive oxygen species (ROS), including superoxides, hydroxyl radicals, and H_2_O_2_ by Zn^2+^. These ROS destroy the cell membrane of bacteria then make reactions with the cytoplasmic constituents until killing the microorganism (Mizielińska et al., 2018). Also, interactions occurring between Zn^2+^ cations and nucleic acids and other biomolecules that have negative charges (e.g., phosphate, disulfide, and sulfhydryl groups pertaining to enzymes) result in the degradation of bacterial walls, membranes, and proteins, ultimately inducing lysis and death in the bacterial cells (Zhang et al., 2010). The outstanding antimicrobial properties of ZnO NPs and the related mechanisms of action have been documented by other researchers (Espitia et al., 2012; Jahed, Khaledabad, Bari, & Almasi, 2017). Nonetheless, when increasing the concentration of ZnO NPs from 5% to 7% of the polymer, no further increase was observed in the inhibition zone, probably due to a fall in the diffusion ability of these nanoparticles (Ngo, Dang, Tran, & Rachtanapun, 2018). Interestingly, the Gram‐positive strain (*S. aureus*) had greater sensitivity in comparison with the Gram‐negative strain (*P. fluorescens*) (Rajesh Kumar, Umar, Kumar, & Nalwa, 2017), supporting the belief that ZnO NPs have antimicrobial effects that are highly dependent on the target organism's cell wall structure (Ngo et al., 2018). Indeed, the diffusion of active antimicrobial agents through the lipopolysaccharide layer of the bacterial cell wall can be limited by the presence of an additional external membrane in Gram‐negative species (Heydari‐Majd, Ghanbarzadeh, Shahidi‐Noghabi, Najafi, & Hosseini, 2019). These findings are in agreement with those of similar studies, in which the relatively less sensitivity of Gram‐negative strains to ROS has been established (Akbar & Anal, 2014; Jebel & Almasi, 2016; Shankar et al., 2015; Sharifalhoseini, Entezari, & Jalal, 2015).

**TABLE 3:** Antimicrobial activity of film samples

Samples	Inhibition zone (mm)	
	*P. fluorescens*	*S. aureus*
G/CNFs 5% (control)	6.21 ± 0.1^a^	6.41 ± 0.13^e^
G/CNFs 5%/ZnO NPs 1%	6.75 ± 0.13^a^	6.85 ± 0.26^e^
G/CNFs 5%/ZnO NPs 3%	7.42 ± 0.11^b^	7.95 ± 0.35^f^
G/CNFs 5%/ZnO NPs 5%	9.75 ± 0.11^c^	10.44 ± 0.44^g^
G/CNFs 5%/ZnO NPs 7%	7.58 ± 0.15^d^	8.43 ± 0.07^h^

Note

Data are expressed as mean ± standard deviation (*n* = 3) and different letters show significant differences at the 5% level in Tukey's test (*p* < .05).

Abbreviations: CNFs, cellulose nanofibers; G, gelatin; ZnO NPs, zinc oxide nanoparticles.

### 3.6 Characteristics of the selected films

#### 3.6.1 Morphology observation by SEM

**FIGURE 3:**
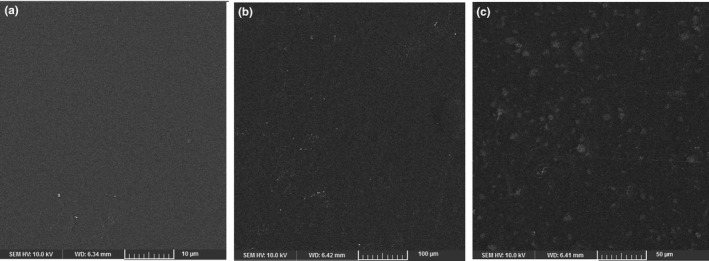
SEM micrographs of the surface of the gelatin films (a) incorporated with CNFs (b) and ZnO NPs (c). CNFs, cellulose nanofibers; ZnO NPs, zinc oxide nanoparticles

In the case of nanocomposites, SEM images can show the distribution of NPs in the matrix, the presence of aggregates and or voids, and the possible orientation of NPs, giving a better understanding of the relations between the physicomechanical and structural properties of the films (Jahed, Khaledabad, Almasi, & Hasanzadeh, 2017). In Figure 3, the SEM images of the surface morphology of the gelatin, G/CNF, and G/CNF/ZnO films are shown. The pure gelatin sample featured a surface that was smooth, compact, and homogeneous without porosity, proving an ordered film structure (Wang, Liu, et al., 2017). As seen, the G/CNF films had homogeneous surfaces with little roughness, lacking air bubbles and cracks; this indicates appropriate compound mixing. The appropriate aggregation and uniform, highly compact structure of these samples are due to the homogeneous distribution of the CNFs in the gelatin matrix, the electrostatic stabilization induced by the CNFs' superficial anionic carboxyl molecules, and the stable bonds formed between the hydrophilic compounds during drying (Alizadeh‐Sani et al., 2018; Wang, Liu, et al., 2017). The same results have been reported for the influence of CNFs on other biopolymer films such as sodium caseinate (Ranjbaryan et al., 2019) and starch (Fazeli, Keley, & Biazar, 2018). The nanocomposite films showed rough and granular surface structures with randomly distributed ZnO NPs. However, significant aggregation was not observed, which indicates that the ZnO NPs were homogeneously distributed through the whole G/CNF matrix. In the SEM images, white dots indicate the occurrence of these ZnO NPs at the polymer surface.

#### 3.6.2 **Fourier transform infrared** (**FT‐IR) spectroscopy**

**FIGURE 4:**
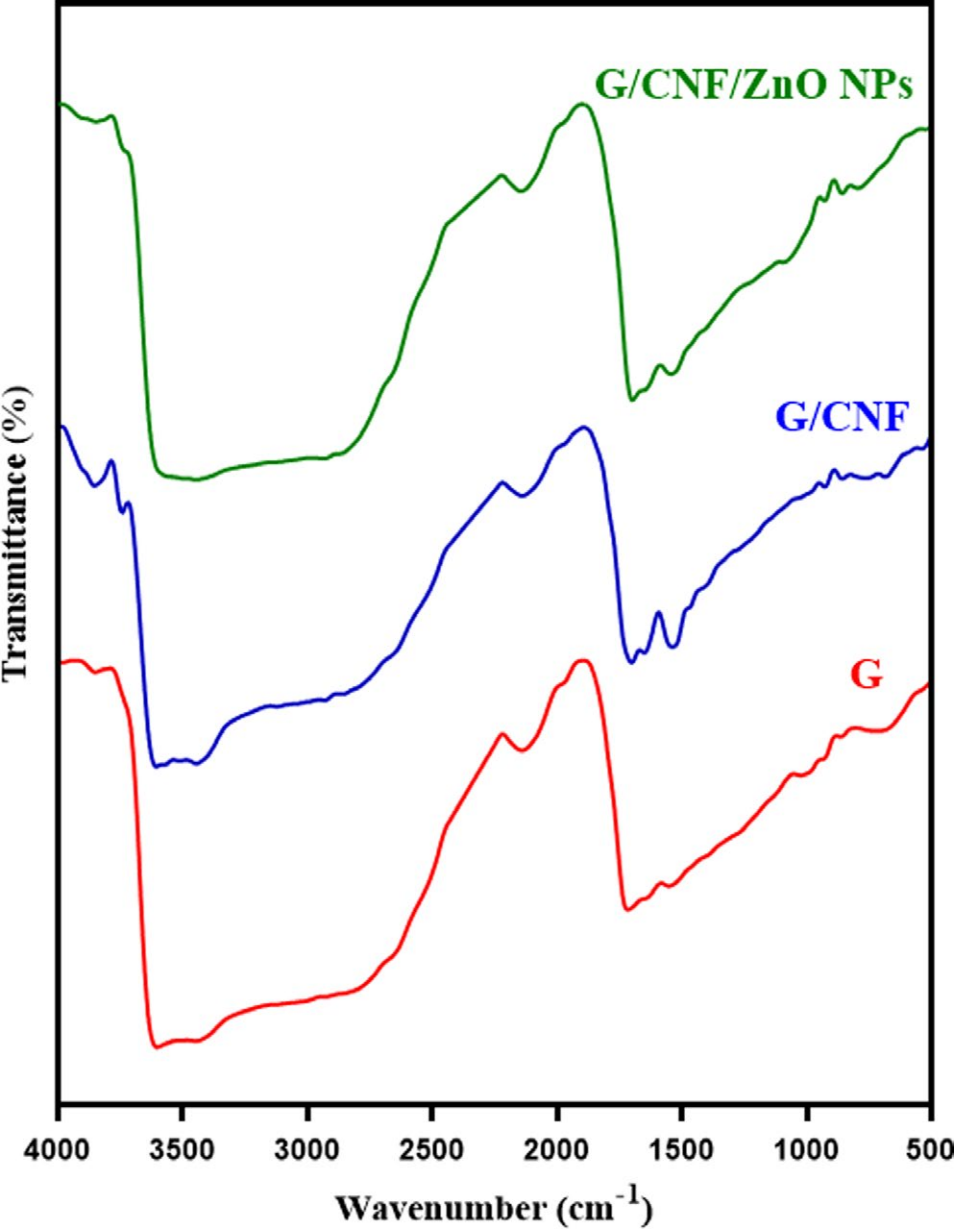
FT‐IR spectra of gelatin films incorporated with CNFs and ZnO NPs

The FT‐IR analysis was conducted to identify the functional groups and the interactions between components of composite film (Figure 4). The peaks situated at the wavenumbers of ~855, ~1,540, ~1,700, ~2,140, ~2,850, and ~3,443 cm^−1^ were approximately found in all films with similar patterns mid slight changes in some peaks. Pure gelatin film displayed a major band at 1,714 cm^−1^, which is the characteristic peak of amide‐I and represents C=O stretching/hydrogen bonding coupled with a COO group (Arfat, Benjakul, Prodpran, Sumpavapol, & Songtipya, 2014; Shankar et al., 2015). The peak at ~1,542 cm^−1^ attributed to amide‐II, arising from the bending vibrations of N–H groups and the stretching vibrations of C‐N groups. The peak near 3,400 cm^−1^ was due to the N‐H stretching of the amide‐A band coupled with hydrogen bonding. The band positioned at the wavenumber of 987–1,041 cm^−1^ corresponding to the interactions between the film structure and the OH group, generally from glycerol added as a plasticizer, was found in all film samples (Alizadeh‐Sani et al., 2018). The other peak around 2,850 cm^−1^ representing C‐H stretching bond (Arfat, Ahmed, Hiremath, Auras, & Joseph, 2017). Amide‐A is related with N‐H stretching vibration of bonded amide groups at ~3,400 cm^−1^. In addition to amide‐A band, in 3,600–3,000 cm^−1^ region, the O‐H stretching vibration of water molecules appeared. Also, the bending vibration of bound water molecules present in the film coupled with the acid carbonyl stretching of amino acid is observed at 1,631 cm^−1^ (Umamaheswari, Sanuja, John, Kanth, & Umapathy, 2015). As it could be seen, some of the peaks are shifted to higher or lower wavenumbers when CNF and ZnO NPs were used. For example, the peak of amide‐I slightly shifted to wavenumbers ~1,700 cm^−1^ and 1,696 cm^−1^ after incorporation of CNF and ZnO NPs, respectively. Like spectral changes in the amide‐I region, a slight change at wavenumbers peaks of amide‐II (shift of 1,520 cm^−1^ peak to 1,536 cm^−1^ and 1,542 cm^−1^ peak) was observed when CNF and ZnO NPs were added, respectively (Mohanapriya, Mumjitha, Purnasai, & Raj, 2016; Shankar et al., 2015). These results implied that the CNF and ZnO NPs were engrafted into the amide bonds of gelatin molecular chains via a conjugation process (Hosseini et al., 2015). By the incorporation of CNF and ZnO NPs, the broadness of the peak between 3,000 and 3,600 cm^−1^ decreased, signifying a decrease in hydrophilicity of gelatin films (Kumar et al., 2019). The N–H stretching of the amide‐A band has transferred from 3,400 cm^−1^ for gelatin film to 3,443 cm^−1^ for G/CNF/ZnO NPs film. Such changes indicated the increased interaction between N‐H groups of the protein chain and nanomaterials, mostly hydrogen bonding in the films (Zubair & Ullah, 2020). Gelatin films display a new peak around 600–900 cm^−1^ which represents the uptake of ZnO by the gelatin (Umamaheswari et al., 2015). Also, two new absorption bands at 926 and 855 cm^−1^ arise from the C‐O‐C stretching at the β‐(1‐4)‐glycosidic linkages which related to cellulose nanofiber structure (Samadani et al., 2019). Incorporation of CNFs and ZnO NPs impressed the intermolecular interaction and molecular association in the gelatin film matrix due to the enhanced multiple covalent bonding and hydrogen bonding (Arfat et al., 2017).

#### 3.6.3 Assessment of inoculated foodborne pathogenic bacteria

**FIGURE 5:**
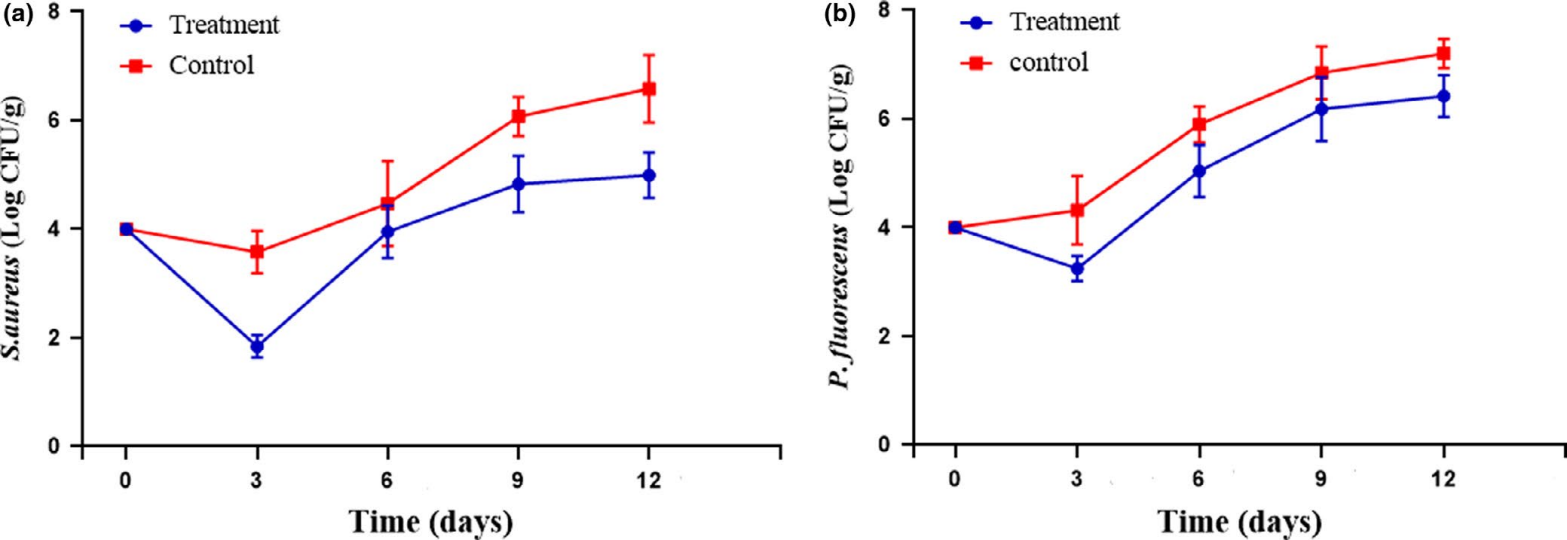
The counts of inoculated *S. aureus* (a) and *P. fluorescens* (b) in chicken fillet samples packaged within sterile transparent polyethylene (control) and the gelatin‐based films incorporated with CNF/ZnO NPs (treatment) during storage at 4°C. Data are presented as mean ± standard deviation (*n* = 3). Each point represents the mean ± *SD*. CNFs, cellulose nanofibers; G, gelatin; ZnO NPs, zinc oxide nanoparticles

The growth rates of *S. aureus* and *P. fluorescens* inoculated in chicken fillets packaged within sterile transparent polyethylene (control) and gelatin‐based films incorporated with CNF/ZnO NPs (treatment) are shown in Figure 5a,b. As a pathogenic agent that is foodborne, *S. aureus* causes various diseases. Food poisoning by *S. aureus* is most commonly related to poultry, egg, red meat, and seafood products (Yuan & Yuk, 2018). According to the results, 3 days after inoculation, the number of *S. aureus* reached 3.58 ± 0.39 and 1.84 ± 0.21 log CFU/g in the control and treatment samples, respectively. This number increased significantly (*p* < .05) across both samples from day 3 onwards during the storage time, though the control had a faster rate of rising in this parameter. At the end of the storage period, the number of bacteria was 6.58 ± 0.62 and 4.99 ± 0.42 log CFU/g in the control and treatment samples, respectively.

Regarding *P. fluorescens*, the results also showed that on day 3, a significantly higher count (4.32 ± 0.63 log CFU/g) was found in the control relative to the treatment sample coated with the G/CNF/ZnO NP film (3.25 ± 0.23 log CFU/g). On day 12, the control sample had a *P. fluorescens* count of 7.20 ± 0.27 log CFU/g, while the treatment had 6.42 ± 0.38 log CFU/g. In both samples, the *P. fluorescens* count increased from day 3 onwards. Generally, the G/CNF nanocomposite film containing 5% ZnO NPs had a significant antimicrobial impact against the pathogenic bacteria (*S. aureus* and *P. fluorescens* bacteria), especially against the Gram‐positive species. Some research suggests that NPs have greater antibacterial activity against Gram‐positive strains (Amjadi et al., 2019; Shankar et al., 2015). Notably, Alizadeh Sani et al. obtained similar findings (Sani, Ehsani, & Hashemi, 2017) using TiO_2_ NPs incorporated in whey protein isolate/CNF nanocomposite films. Resistance of bacteria to the surrounding environment is provided by the cell wall and membrane, with their components producing various absorbency pathways for NPs in both Gram‐positives and Gram‐negatives (Lesniak et al., 2013). In Gram‐negatives, the cell wall's outer membrane is constituted by lipopolysaccharide (LPS), lipoproteins, and phospholipids, usually making it thicker than the peptidoglycan layer of the Gram‐positive cell wall; this gives rise to greater resistance to the passage of substances and only permeates the entry of macromolecules. These structural properties of the Gram‐negative bacterial cell inhibit lipid peroxidation by ROS produced by ZnO nanomaterials and reduce their susceptibility to the ZnO attack (Kumar et al., 2017). The LPS is responsible for ion flow regulation and prevents ZnO NP adhesion to the bacterial cell wall (Yu et al., 2014). In contrast, the cell wall of Gram‐positive strains has a thin peptidoglycan layer along with teichoic acid; the porous nature of this cell wall allows entrance of molecules from the environment, leading to possible death via damage to the cell membrane. Moreover, the more negative surface charge of the Gram‐positive cell wall can cause the attraction of the positively charged NPs to the bacteria (Sarwar, Katas, Samsudin, & Zin, 2015; Wang, Hu, & Shao, 2017).

## 4 CONCLUSION

In the present research, we introduced an eco‐friendly, gelatin‐based, active nanocomposite film containing CNFs and ZnO NPs. Gelatin‐based films are suitable carriers for reinforcement fillers (e.g., CNFs) and antimicrobial substances (e.g., ZnO NPs). Based on physicomechanical (WVP, WS, MA, MC, TS, YM, and EAB) and antibacterial characterization, the optimal concentrations of CNFs (5%) and ZnO NPs (5%) for addition to the gelatin matrix were ascertained. The TS and YM of the G/CNF film were significantly higher relative to the pure gelatin film. The G/CNF film had significantly improved resistance against water vapor (i.e., less WVP) relative to the gelatin film. Considering the results of the disk diffusion test and bacterial count, the ZnO NPs showed antibacterial effects against both Gram‐positive and Gram‐negative species, particularly the former. In conclusion, antimicrobial active packaging films containing ZnO NPs can be used as a beneficial solution for preserving the quality and safety of fresh meat products.
